# Analysis of 953 Human Proteins from a Mitochondrial HEK293 Fraction by Complexome Profiling

**DOI:** 10.1371/journal.pone.0068340

**Published:** 2013-07-23

**Authors:** Hans J. C. T. Wessels, Rutger O. Vogel, Robert N. Lightowlers, Johannes N. Spelbrink, Richard J. Rodenburg, Lambert P. van den Heuvel, Alain J. van Gool, Jolein Gloerich, Jan A. M. Smeitink, Leo G. Nijtmans

**Affiliations:** 1 Radboud Proteomics Centre, Department of Laboratory Medicine, Laboratory of Genetic Endocrine and Metabolic Disorders, Radboud University Medical Centre, Nijmegen, The Netherlands; 2 Nijmegen Centre for Mitochondrial Disorders, Department of Pediatrics, Radboud University Medical Centre, Nijmegen, The Netherlands; 3 Welcome Trust Centre for Mitochondrial Research, Medical School, Newcastle University, Framlington Place, Newcastle upon Tyne, United Kingdom; 4 Institute of Biomedical Technology & Tampere University Hospital, University of Tampere, Tampere, Finland; Moffitt Cancer Center, United States of America

## Abstract

Complexome profiling is a novel technique which uses shotgun proteomics to establish protein migration profiles from fractionated blue native electrophoresis gels. Here we present a dataset of blue native electrophoresis migration profiles for 953 proteins by complexome profiling. By analysis of mitochondrial ribosomal complexes we demonstrate its potential to verify putative protein-protein interactions identified by affinity purification – mass spectrometry studies. Protein complexes were extracted in their native state from a HEK293 mitochondrial fraction and separated by blue native gel electrophoresis. Gel lanes were cut into gel slices of even size and analyzed by shotgun proteomics. Subsequently, the acquired protein migration profiles were analyzed for co-migration *via* hierarchical cluster analysis. This dataset holds great promise as a comprehensive resource for *de novo* identification of protein-protein interactions or to underpin and prioritize candidate protein interactions from other studies. To demonstrate the potential use of our dataset we focussed on the mitochondrial translation machinery. Our results show that mitoribosomal complexes can be analyzed by blue native gel electrophoresis, as at least four distinct complexes. Analysis of these complexes confirmed that 24 proteins that had previously been reported to co-purify with mitoribosomes indeed co-migrated with subunits of the mitochondrial ribosome. Co-migration of several proteins involved in biogenesis of inner mitochondrial membrane complexes together with mitoribosomal complexes suggested the possibility of co-translational assembly in human cells. Our data also highlighted a putative ribonucleotide complex that potentially contains MRPL10, MRPL12 and MRPL53 together with LRPPRC and SLIRP.

## Introduction

Protein-protein interactions are essential for many different cellular processes. Perturbed protein-protein interactions can have strong negative effects on cell viability, which in turn may have devastating effects in an organism. This is exemplified by the severe clinical syndromes that are associated with assembly defects of the mitochondrial oxidative phosphorylation (OXPHOS) complexes [1], [2]. Other examples of disease that involve gained, lost or perturbated protein-protein interactions are Charcot-Marie-Tooth disease, Alzheimer's disease, Huntington's disease, multiple acyl-CoA dehydrogenation deficiency, MCAD deficiency, hereditary spastic paraplegia, and pathogen-host interactions [3]–[5]. Cataloguing of protein-protein interactions not only contributes to the fundamental understanding of cellular biology but also provide insight into the pathogenic mechanisms that underlie disease. Ultimately, such data can be used to develop pharmaceutical interventions in selected cases *via* targeted disruption of protein-protein interactions by antibodies, peptides, or even small molecules [3]. It is therefore important for fundamental-, clinical-, and pharmaceutical-research to unravel protein-protein interactions.

**Figure 1:**
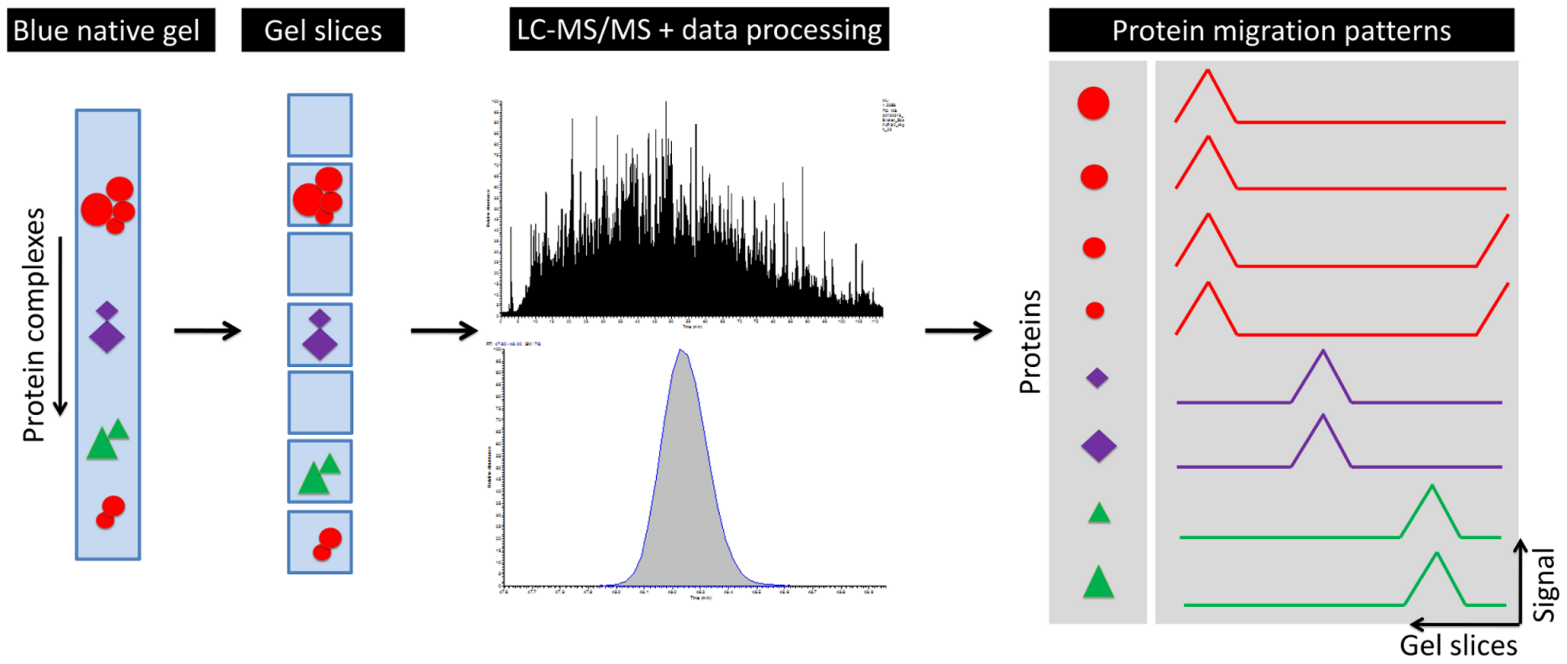
Schematic overview of the complexome profiling approach. Protein complexes are separated according to size by blue native gel electrophoresis after which the gel lane is cut into gel slices at even distance. Each gel slice is separately processed by tryptic in-gel digestion and subsequently analyzed by liquid chromatography combined with online tandem mass spectrometry. In the final steps, the peptide identifications with according relative abundance from each individual LC-MS/MS analysis are combined to reconstruct the migration profile for each protein that span the complete length of the blue native separation. Please note that two subunits of the large red complex were also available as a smaller complex in this example to include proteins that form multiple complexes.

Blue native gel electrophoresis (BNE) has been developed to study native protein complexes [6]–[8]. In this procedure, protein complexes are solubilised in their native state, decorated with the charged dye Comassie Blue, and separated by size using electrophoresis in gradient acrylamide gels. Large-scale analysis of protein-protein interactions by BNE was previously performed by two dimensional blue native/ sodium dodecyl sulphate polyacryl amide gel electrophoresis (2D BN SDS-PAGE) combined with mass spectrometry. Protein complexes are separated in a first dimension BNE followed by a second denaturing SDS-PAGE step to resolve protein complexes into their respective subunits. Stained protein spots are excised and submitted to tryptic digestion to identify each protein *via* its proteolytic peptides by mass spectrometry [9]. We have reported a method that omits the second dimension SDS-PAGE and spotpicking-based mass spectrometric identification of proteins by direct analysis of fractionated BNE gel lanes by liquid chromatography – tandem mass spectrometry [10]. This method applies labelfree semi-quantitative shotgun proteomics to blue native gel lanes that are cut into gel slices of equal size. The acquired mass spectrometry data is used for protein identification and to determine the relative abundance of each respective protein in the gel slices to cover the entire blue native separation length. This information is then used to assemble the *in-silico* protein migration profiles. The outline of this method is schematically shown in figure 1. Following its introduction, different groups have successfully applied the methodology to study protein-protein interactions [11]–[14]. By the application of hierarchical clustering Heide *et al* [11] extended this method to a true bottom-up proteomics approach coined complexome profiling.

One of the advantages of complexome profiling over the classical 2D BN SDS-PAGE method is that the electrophoretic profiles are stored in a data matrix of proteins (rows) with gel slices (columns) where each cell contains the respective relative abundance. This allows the unambiguous analysis of protein co-migration by computational methods such as protein correlation profiling [10], [15], [16] and clustering methods [11], [12], [14]. Once acquired, the dataset can be used to identify potential *de novo* protein-protein interactions or drive validation of predicted protein-protein interactions from e.g. affinity purification – mass spectrometry (AP-MS) experiments. The latter application is of particular interest as the mass spectrometric identification of co-purified proteins on itself does not provide physicochemical information about the actual interactions themselves or the size of the complexes [11].

In this paper we present the first complexome profiling dataset from human cells which can be used by researchers to support alleged protein-protein interactions, identify novel protein-protein interactions, or to prioritize candidate protein interactors. A small subset of the protein profiles generated *via* different quantitation methods was previously used to deliver the proof-of-principle for the approach [10] and identified at least two complex I assembly chaperones [10], [17], [18]. Following further advances in data processing methods (such as peptide selection based on profile similarity rather than intensity to construct protein profiles), we here describe the complete dataset of 953 protein migration profiles from a mitochondrial HEK293 fraction in two acrylamide (AA) gradient gels of 4–12% and 5–15% AA. Hierarchical clustering (HCL) was used to order the protein migration profiles for ease of use, which is also provided as a supplementary data with supporting evidence from STRING [19]–[21] and DAVID [22], [23] for protein relationships within each cluster.

Four of the HCL clusters were of particular interest as they contained proteins of the 28 S and 39 S mitochondrial ribosome (mitoribosome) which has not been analyzed by BNE thus far. Recent publications reported interactions between many different proteins and the mitoribosome based on AP-MS results [24]–[27]. To demonstrate the potential of our dataset to underpin AP-MS results we have analyzed the protein migration profiles from recently reported mitoribosome-interacting proteins for co-migration with mitoribosomal subunits.

## Methods

### Sample preparation

HEK293 cells grown in DMEM (Biowhitaker) supplemented with 10% (v/v) FCS and 1% (v/v) penicillin/streptomycin were harvested at ∼80% confluency using PBS. Cells were disrupted by pottering in isotonic buffer and a mitochondrial enriched fraction was obtained by centrifugation according to Vogel [28]. Native protein complexes were extract from the mitochondria-enriched pellet by resuspension of the pellet in 200 µl ACBT (1.5M aminocaprioc acid, 75 mM Bis-Tris pH 7.0) and 22 µl of 20% (w/v) *n*-dodecyl b-d-maltoside(approximately 1 gr/gr protein). The suspension was incubated for 10 minutes on ice to solubilize proteins and subsequently centrifuged at 10 000×g for 25 minutes at 4°C. The concentration of solublilized proteins in the supernatant was determined using the MicroBCA protein assay kit (Pierce) prior to addition of BN sample buffer. BN gels (4–12% and 5–15% acryl amide gradient gels) were cast using Bio-Rad mini PROTEAN® II systems in combination with 1.5 mm spacers. Both gels were loaded with 80 µg of protein per lane and run till the dye front reached the end of each respective gel.

The resulting gel lanes were cut into 24 gel slices at even distance and each gel slice was subjected to *in-gel* tryptic digestion essentially according to Heide [11]. Briefly, gel slices were cut into 1×1 mm cubes and transferred to a fresh 96-well plate. The gel particles were washed successively three times with 50 mM ammonium bicarbonate (AHC), 50% (v/v) acetonitrile (ACN) and 100% (v/v) ACN for 20 minutes under gentle agitation. Gel particles were swelled in 10 mM dithiotreitol and incubated for 30 minutes at 56°C to reduce protein disulfide bonds. After removal of the reduction buffer, gel particles were shrunk in ACN for 20 minutes at room temperature under gentle agitation. Alkylation of reduced cysteine residues was performed by incubating the gel particles for 30 minutes in alkylation buffer (50 mM chloroacetamide in 50 mM AHC) at room temperature in the dark. Following the alkylation step, gel particles were washed successively with 50 mM AHC and ACN prior to the addition of 50 µl 12.5 ng/µl trypsin (Promega: V511C) and overnight protein digestion at 37°C. Proteolytical peptides in the digestion buffer were recovered by transfer of the buffer solution to a fresh 96-well plate. Remaining proteolytical peptides in the gel pieces were recovered by shrinking the gel particles in ACN for 30 minutes at room temperature under gentle agitation and subsequent transfer of the peptide-containing ACN solution to the 96-well plate. The combined peptide extracts were subjected to *in vacuo* centrifugation to remove acetonitrile. Subsequently, stop and go elution (STAGE) tips [29] were used to desalt and concentrate the peptide mixtures prior to LC-MS/MS measurements.

### Liquid chromatography – tandem mass spectrometry

Duplicate measurements for each gel slice were performed by nanoflow reversed-phase C18 liquid chromatography (Agilent 1100 series) coupled online to a 7T linear ion trap Fourier-Transform ion cyclotron resonance mass spectrometer (LTQ FT, Thermo Fisher Scientific) [30], [31]. Chromatographic separations were performed using a 15 cm long×100 µm ID fused silica electrospray emitter (New Objective, PicoTip Emitter, FS360-100-8-N-5-C15) packed *in-house* at 100 bar with ReproSil-Pur C18AQ 3 µm 140 Å resin (Dr. Maisch) resuspended in methanol. Proteolytical peptides were loaded directly onto the analytical column using 0.5% (v/v) acetic acid at a flow rate of 600 nl/min. A linear gradient of 12–46% acetonitrile (ACN) with 0.5% (v/v) acetic acid as ion pair reagent was used to gradually elute peptides from the column at a flow rate of 300 nl/min. Following each analysis, the column was washed for 10 minutes with 80% ACN at 600 nl/min and conditioned using 0.5% acetic acid for 10 minutes at 600 nl/min. Intermittent blank injections were performed to minimize carry-over effects between samples.

The mass spectrometer was operated in positive ion mode and programmed to acquire a single full MS survey scan in the ICR cell with up to four data dependent collision induced dissociation (CID) fragmentation spectra in parallel by the linear ion trap. The mass spectrometer was tuned on MRFA peptide (*m/z* 524) at 300 nl/min and calibrated using caffeine (*m/z* 195), MRFA (*m/z* 524) and Ultramark 1621 (*m/z* 1122, 1222, 1322, 1422, 1522, 1622, 1722 and 1822) standards from the MSCAL5 ProteoMass™ LTQ/FT-Hybrid ESI Positive Mode CalMix kit (Sigma Aldrich). The ICR cell precursor scans were performed using a single microscan at 50 000 resolving power (FWHM at *m/z* 400) on 1E6 ions or 500 ms maximum injection time (whichever came first). Data dependent acquisition of MS/MS spectra by the linear ion trap used a single microscan at normal scan speed. The automatic gain control was set to 1E4 ions for MS/MS with 400 ms maximum injection time. Collision induced dissociation of precursor ions was performed at 27% normalized collision energy for 30 msec and activation Q = 0.250. An isolation width of 3 Th was used to trap and isolate precursor ions for MS/MS experiments. Dynamic exclusion was enabled to minimize re-analysis of precursor ions (list size: 500, exclusion time: 300 sec, 1 repeat count, 1.5 amu precursor mass tolerance).

### Database searches

Raw mass spectrometry data was converted into Mascot search engine compatible peak lists by ExtractMsn (Thermo Fisher Scientific) and an *in-house* developed Perl script. Mascot database searches were performed against the *Homo sapiens* RefSeq (release 44) database [32], [33] with added sequences of known contaminant proteins (e.g. trypsin). The decoy database used for false discovery rate (FDR) validation contained the reversed protein sequences. Mascot (Matrix Science) [34] database searches were performed with 15 ppm and 0.5 Da mass tolerance for precursor ions and fragment ions, respectively. Carbamidomethylation (Cys) was specified as fixed modification whereas acetylation (protein N-terminus) and oxidation (Met) were selected as variable modifications. A single missed tryptic cleavage was tolerated. Mascot database search results were exported as peptide XML files and validated according to Weatherly [35] to achieve a maximum 1% FDR at the protein level.

### Quantitation and generation of protein abundance profiles

**Figure 2:**
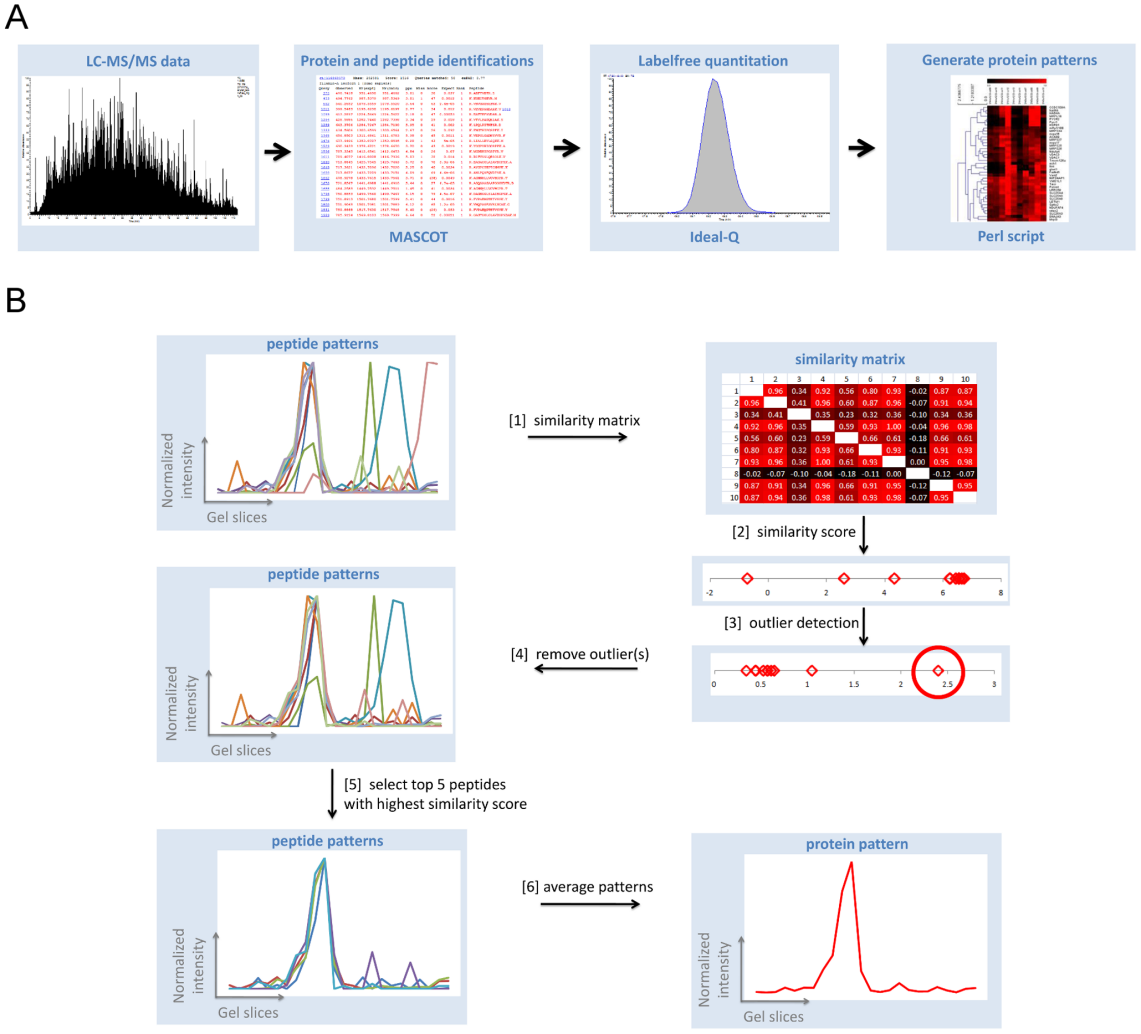
LC-MS/MS data processing and protein profile generation. **Figure 2A** provides an overview for the main steps in LC-MS/MS data processing. The acquired mass spectrometry data is used to identify peptides and protein for each gel slice via database searches using the Mascot search engine. Resulting peptide identifications together with the LC-MS data are used as input for the label-free quantitation by the Ideal-Q software that integrates the chromatographic peak surfaces for each peptide from respective extracted ion currents. Quantitative information from all LC-MS/MS analyses is then used to determine the relative abundance for each peptide in every gel slice. The final step in data processing uses the peptide profiles to reconstruct the migration profile for each individual protein in the blue native gel separation. Details for the reconstruction of the protein migration profiles are shown in **figure 2B** with data for the cytochrome *c* oxidase subunit 6 C protein. First, all peptide profiles of a protein are used to generate a similarity matrix that contains the Pearson's correlation coefficients between each peptide profile. This information is then used to calculate a similarity score for each peptide which is defined as the sum of all the Pearson's correlation coefficients for the peptide from the similarity matrix. The next step uses a Grubb's outlier test on the calculated similarity scores to discard peptide profiles that poorly correspond with the general peptide migration profile. Finally, the peptide migration profiles are ranked in descending order of their similarity score of which the 5 highest scoring peptide profiles are used to construct the protein migration profile by averaging.

Quantitative information was extracted for valid protein identifications as integrated ion current chromatograms of respective peptides by IDEAL-Q software [36] using mzXML files generated by the ReadW software tool [37]. Parameters used for IDEAL-Q data processing specified 30 ppm mass tolerance, a minimal Mascot ion score of 25, and unique peptides only. Quantitative data were exported as peptide level results. An *in-house* developed Perl script was used to generate protein abundance profiles from IDEAL-Q output data. Figure 2 illustrates the different processing steps that were used to generate protein abundance profiles. For each protein, the integrated ion current chromatograms for all identified peptides were extracted from each gel slice. The peptide abundance profiles were scaled between 0 and 1 to ensure equal weight of each peptide abundance profile. Next, a similarity matrix was constructed from all peptide abundance profiles for each protein using Pearson's correlation coefficients. Similarity scores for all peptides of a protein were calculated as the sum of all Pearson's correlation coefficients from the similarity matrix. Aberrant peptide abundance profiles for each protein were excluded from further processing by a Grub's outlier test (with significance *p*>0.9) and remaining peptide abundance profiles were ranked by descending similarity score. Finally, the protein abundance profiles were generated as the average profile from the top 5 peptide abundance profiles with highest similarity scores. Protein abundance profiles from duplicate gel slice measurements were averaged for each acryl amide gradient. Finally, a core dataset of 953 protein abundance profiles was compiled that consists of proteins that were detected in both acryl amide gradients.

### Metadata

Protein identifiers, gene ontology information, and official gene symbols were obtain *via* DAVID [22] and ProteinCenter (Thermo Fisher Scientific). MITOP2 [38] was used to identify mitochondrial reference proteins and to map predicted mitochondrial proteins by the MITOP2 SVM algorithm. For each experiment, the number of peptides and the average Pearson's correlation coefficient from the selected top 5 peptide abundance profiles per protein are listed. Additionally, the Pearson's correlation coefficients for duplicate protein abundance profiles from each acryl amide gradient are provided as additional quality control measure. Mass ranges as well as the average mass of each gel slice were calculated using the known molecular masses of OXPHOS complexes [39].

### Hierarchical clustering analyses

Hierarchical clustering for all mitochondrial protein abundance profiles was performed by the multiple experiment viewer (MEV) software [40]. Uncentered Pearson's correlation coefficients were used as distance metric for clustering in combination with complete linkage distance and optimized leaf order. Clusters were defined in MEV software using an arbitrary distance cut-off of 0.540892 for further analysis. Proteins from each cluster were analyzed by STRING and DAVID tools to mine available data sources for known protein relationships within clusters. To investigate the rate at which proteins cluster together by mere chance we compared the hierarchical clustering results for a set of 16 known mitochondrial complexes from the dataset with results obtained from 10 decoy matrices that contained shuffled abundance profiles. Here, the average number of clusters that contained two or more subunits as well as the average number of clustered proteins for each complex were used to assess the hierarchical clustering results.

Co-migration of mitoribosomal proteins with previously reported interactors from affinity purification – mass spectrometry experiments were analyzed by hierarchical clustering. Proteins reported by Rorbach [26], Richter [27], and He [24], [25] together with all detected mitoribosomal proteins were used to select protein abundance profiles for further analysis. Here, exclusively relevant gel slices for each of the four mitoribosomal complexes detected in the initial hierarchical clustering analysis were used to increase selectivity and sensitivity of the analysis for each respective complex. Hierarchical clustering was performed using Euclidian distance metric to minimize false positives from low abundance data.

## Results

### Dataset description and characteristics

**Figure 3:**
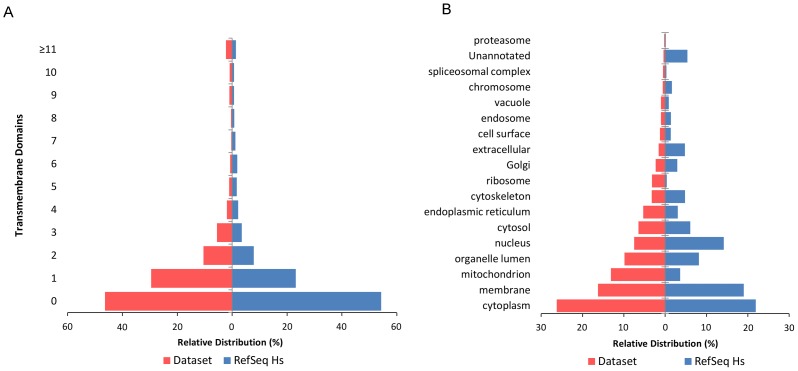
Distributions of transmembrane domains and cellular compartments of proteins in the dataset. ****Figure** **3A**** shows the tornado diagram of transmembrane domains in proteins from both the complexome profiling dataset and the complete RefSeq *Homo sapiens* database. A relative enrichment of 8% more proteins with transmembrane domains was observed for the complexome dataset with respect to the RefSeq Hs database. The tornado diagram for the cellular compartment distribution of proteins in **figure** **3B** show an enrichment of mitochondrial proteins in our dataset compared to the RefSeq Hs database. Please note that proteins may have multiple cellular compartment GO annotations.

A combined total of 118760 MS/MS spectra were mapped to 3981 proteins or 1766 protein identification groups. Proteins were identified at ≤1% FDR with an average of 9 non-redundant peptides and an average summed Mascot ion score of 417 (min: 43, max: 5765). Minimum Mascot ion score thresholds to achieve 1% protein FDR or better were determined to be 50, 27, 21, 21, 19 and 16 for proteins with coverage of 1, 2, 3, 4, 5 and ≥6 peptides, respectively. Peptides were identified with an average Mascot ion score of 41 (min: 16, max: 152) and an average precursor ion mass error of 4.85±3.03 ppm. Analysis of the HEK293 mitochondrial fraction by complexome profiling using two acrylamide gradients resulted in a dataset of 953 proteins where migration profiles could be constructed for both the 4–12% and 5–15% acrylamide (AA) gels. Here, the use of two AA gradient gels allows for cross-validation of protein co-migration and helps to distinguish between protein complexes of similar size. Valid protein and peptide identification data is available from file S1. For the set of 953 proteins we have analyzed the molecular weight, isoelectric point and hydrophobicity (GRAVY) distributions with respect to the RefSeq Hs database to identify possible enrichment of protein classes based on these physicochemical properties. Results shown in file S2 indicate no selective enrichment of proteins based on molecular weight or isoelectric point as both relative distributions are virtually identical to those of the RefSeq Hs database (Pearson's *r* = 0.991 and *r* = 0.994, respectively). However, the hydrophobicity distribution of proteins in our dataset shows a slight shift towards more hydrophobic proteins. This might be explained by the relative high abundance of mitochondrial membrane proteins in combination with the protein extraction and electrophoresis methods that were optimized for the analysis of membrane proteins. This is supported by a significant enrichment for proteins with one or more transmembrane domains with respect to the RefSeq Hs database (53.62% versus 45.66%, FDR adjusted *p*-value  = 7.35E-7). The distribution of transmembrane domains for proteins in the dataset as well as the RefSeq Hs database is shown in figure 3A. In addition, we compared the hydrophobicity distribution of proteins in our dataset to that of mitochondrial proteins in the RefSeq Hs database (file S2). Interestingly, the hydrophobicity distribution of proteins in our dataset is more identical to that of the subset of mitochondrial proteins in the RefSeq Hs database compared to the hydrophobicity distribution of all RefSeq Hs proteins (Pearson's *r* = 0.993 versus *r* = 0.927, respectively).

Because a mitochondria enriched fraction was used in this study, the dataset not only contains mitochondrial proteins but also provides migration profiles of proteins that reside in other cellular organelles. Unfortunately, the mitochondrial localization of many proteins is still unknown, and we therefore included information from the MITOP2 database [38] besides GO annotation [41] to annotate predicted mitochondrial proteins. Proteins included in the MITOP2 mitochondrial reference dataset as well as predicted to be mitochondrial by the MITOP2 support vector machine approach were also considered as mitochondrial proteins in this work. Based on GO annotations, proteins in our dataset predominantly localized to mitochondria (437 proteins), nucleus (250), cytosol (215 proteins), and endoplasmic reticulum (176 proteins). The relative distribution of proteins over cellular compartments is shown in figure 3B. Mitochondrial proteins were significantly enriched in our dataset compared to the RefSeq Hs database based on GO annotations (45.95% versus 8.98%, FDR adjusted *p*-value  = 5.65E–18).

The protein profiles dataset is provided as file S3 for download and contains the protein abundance profiles with according meta-information for BNE AA gradients. Meta-information includes protein description, identifiers (protein Gi code, NCBI RefSeq ID, Uniprot ID, official gene symbol), and cellular localization (Gene Ontology, MITOP2 reference set, MITOP2 SVM prediction). Additional information is provided that can be used to assess the robustness of the protein migration profile. These include the number of peptides used to reconstruct the protein migration profile, average Pearson's correlation coefficient of the peptide profiles with respect to the average (protein) profile, and the Pearson's correlation coefficient for duplicate experiments from the same acrylamide gradient gel.

### Co-migration of subunits from known complexes

Out of the 953 proteins, 209 proteins could be assigned to 24 known heteromeric complexes based on protein description or online information resources [5], [11], [42]. Subunits from protein complexes should theoretically co-localize at the respective migration distance of the protein complex in blue native gels. This was observed for the majority (88%) of subunits from known protein complexes in the dataset as shown in detail in file S4 and summarized in table 1. However, some subunits (12%) of several complexes did not co-localize as expected. This could be caused by solubilization or electrophoresis effects since data from both acryl amide gradients generally show similar profiles for most of these subunits. Nevertheless, the majority of subunits from known protein complexes co-localized as expected in both gradients, confirming the reproducibility of the approach.

**Table 1:** Co-localization and hierarchical clustering details for known protein complexes in the dataset.

		Co-localized subunits		
Protein complex	Detected subunits	4–12% AA	5–15% AA	Proteins clustered together by HCL
Complex I – NADH dehydrogenase	28	28	28	HCL27: 22 (79%)
Complex II – Succinate dehydrogenase	2	2	2	HCL 14: 2 (100%)
Complex III – Cytochrome bc1 complex	7	7	7	HCL 26: 6 (86%)
Complex V – ATP synthase	11	11	11	HCL 32: 10 (91%)
TCP containing chaperone complex	8	8	8	HCL27: 7 (100%)*
Isocitrate dehydrogenase	4	4	4	HCL 25: 4 (100%)
propionyl-CoA carboxylase	2	2	2	HCL 27: 2 (100%)
Prohibitin complex	2	2	2	HCL 28: 2 (100%)
Proteasome	7	7	7	
Integrin complex	6	6	6	
V-type proton ATPase: V1 part	3	3	3	
V-type proton ATPase: V0 part	3	3	3	
beta-hexosaminidase	2	2	2	
2-oxoisovalerate dehydrogenase	2	2	2	HCL 14: 2 (100%)
electron transfer flavoprotein	2	2	2	HCL 15: 2 (100%)
Trifunctional enzyme	2	2	2	HCL 25: 2 (100%)
28S mitochondrial ribosome	24	23	23	HCL 28: 13 (54%), HCL 29: 3 (13%), HCL 31: 6 (25%)
Complex IV - Cytochrome C oxidase	7	6	6	HCL 21: 5 (71%)
dolichyl-diphosphooligosaccharide--protein glycosyltransferase	6	5	5	
39S mitochondrial ribosome	29	22	22	HCL1: 17 (59%), HCL23: 3 (10%)
40S Ribosome	16	13	10	
2-oxoglutarate dehydrogenase	3	2	3	0%
60S Ribosome	32	19	26	
Pyruvate dehydrogenase	3	0	0	0%

This table shows the number of available subunits in the dataset for each annotated protein complex with the according number of subunits that co-localize in each acryl amide gradient. Presented here are also the number of subunits that reside within the same cluster(s) for each mitochondrial protein complex from the hierarchical clustering analysis of the data. *Of the eight TCP complex subunits seven were predicted to be mitochondrial and were included in the HCL analysis.

### Hierarchical cluster analysis of mitochondrial protein profiles

**Figure 4:**
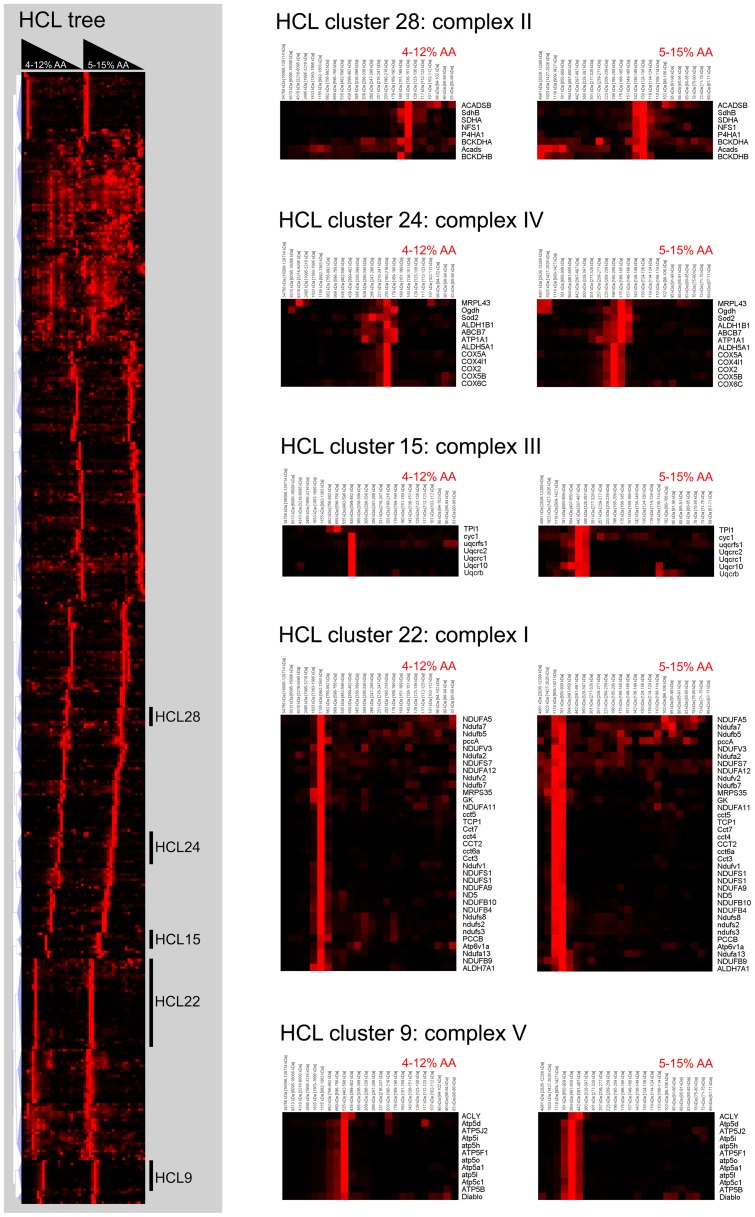
Representative hierarchical clustering results of mitochondrial protein profiles. Profiles from proteins with mitochondrial annotation (MITOP2 reference dataset or Gene Ontology) or mitochondrial prediction (MITOP2 SVM) were subjected to hierarchical clustering (uncentered Pearson's correlation metric, optimized leaf order, and complete linkage distance). Five representative clusters are shown that contain the five mitochondrial oxidative phosphorylation system (OXPHOS) complexes. Clusters that are referenced in the text are indicated in the HCL tree with numbers that correspond with cluster numbers defined by the MEV software.

Some of the mitochondrial proteins in the dataset were expected to form yet unknown protein complexes. Since the protein profiles were stored in a data matrix, the dataset allowed for the use of computer algorithms in the analysis of protein co-migration profiles. We applied hierarchical cluster analysis (HCL) to categorize the dataset for ease of use, similar to the approach of Heide [11]. Figure 4 shows the HCL analysis results which are provided as MS Excel file (file S5). The HCL_clusters.xls file contains the large high-resolution version of the HCL tree, individual clusters with according HCL and STRING images, as well as an index to look up proteins of interest within clusters. File S6 is the MEV analysis file that contains both the HCL analysis as well as the analyzed data matrix for ease of use. The MEV software is freely available [40] and should be used to open this analysis file. As quality control measure for the HCL we summarized the results for known complexes in table 1. Ideally, one would expect that all subunits of a given complex would group together in a single cluster. This holds true for the majority of subunits listed in table 1. Subunits of complexes that did not group together in the same cluster are explained by the presence of those particular subunits at higher relative abundance in another gel slice. Nevertheless, nearly all subunits of the mitochondrial complexes in table 1 (except for the 2-oxoglutarate dehydrogenase and pyruvate dehydrogenase complexes) were clustered correctly.

To investigate the rate at which proteins cluster together by mere chance we constructed 10 randomized matrices in which the abundance values were shuffled for each respective AA gradient gel. For each randomized matrix we performed the same hierarchical cluster analysis that was used for the “real” dataset. To determine the rate at which proteins cluster together we focussed on the 16 known mitochondrial complexes described in table 1 and calculated the average number of clusters that contain two or more subunits from each complex as well as the average number of clustered proteins. Results in file S7 show that subunits from complexes in the randomized data cluster together at a much lower rate in comparison to the real dataset. On average, 12 subunits from three complexes in the randomized data clustered together versus 108 subunits from 14 complexes in the original dataset. The co-clustering of subunits in the randomized dataset concerned proteins that showed multiple features in their abundance profiles. Complex abundance profiles likely increase the probability for these proteins to cluster together with respect to proteins that are part of only a single protein complex.

**Figure 5:**
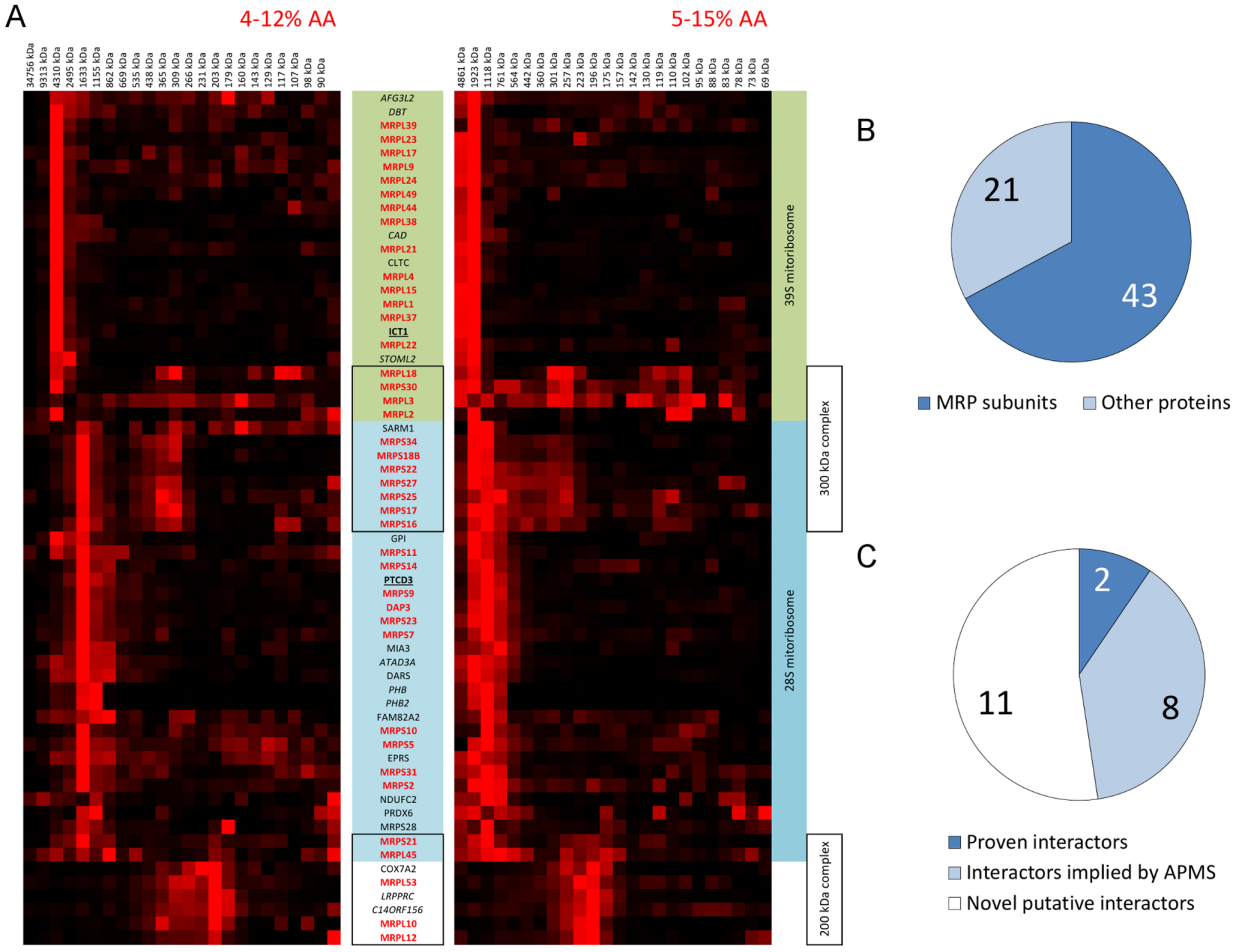
Identified mitochondrial ribosomal complexes by hierarchical clustering. Subunits of the mitoribosome were predominantly found in 4 distinct complexes by the HCL analysis as shown in **figure 5A**. Known subunits of the mitoribosome are bold red whereas proven interactors are in black and bold underlined. Proteins previously co-purified with mitoribosomal subunits are italic. The majority of detected MRPL subunits co-migrate in a complex of about 3 MDa in size which is referred to as the 39 S mitoribosome complex in this paper. Besides MRP subunits, six other proteins showed co-migration of which ICT1 is a proven interactor of the mitoribosome. The remaining three proteins DBT, STOML2, and CAD have previously been reported to co-purify with mitoribosomal proteins in affinity purification – mass spectrometry studies. Similar to MRPL proteins, all but one of the 28 S mitoribosomal MRPS subunits showed to co-migrate in a complex of about 1.6 MDa together with 12 other proteins that include the known mitoribosome interactor PTCD3. Four of the remaining 11 proteins have been reported to co-purify with mitoribosomal subunits in affinity purifications.This complex is referenced in the text as the 28 S mitoribosome complex.Figure 5A also shows a smaller complex of about 300 kDa in size that includes 8 MRPS and 3 MRPL subunits of the mitoribosome together with SARM1.Finally, another complex of about 200 kDa in size was detected that appears to consist of five mitoribosomal proteins together with LRPPRC, C14ORF156 (SLIRP), and COX7A2. **Figure 5B** shows the distribution of MRP subunits versus non-MRP proteins detected in any of the four complexes and **Figure** ** **5C** shows the distribution of the 21 non-MRP proteins in three classes: proven interactors, proteins co-purified with mitoribosomal proteins in affinity purification – mass spectrometry studies, and proteins that have thus far not been described in literature related to the mitochondrial ribosome.

Four clusters from the HCL analysis were of particular interest as they contained multiple 28 S and 39 S subunits of the mitoribosome which are shown combined in figure 5 and table 2. Interestingly, the 28 S and 39 S HCL clusters contained 19 proteins of which eight were recently reported to associate or interact with the mitoribosome [24]–[27]. The 28S subunits were found to co-migrate with SARM1, GPI, PTCD3, MIA3, ATAD3A, DARS, PHB, PHB2, FAM82A2, EPRS, PRDX6, and NDUFC2. Of these twelve proteins, four are reported interactors of the mitoribosome: PTCD3, ATAD3A, PHB, and PHB2 [24]–[27]. Likewise, the 39 S cluster contained six co-migrating proteins of which four were previously found to co-purify with mitoribosomes: AFG3L2, DBT, ICT1, and STOML2 [24]–[27]. Interestingly, the 28 S HCL cluster also showed the presence of a smaller complex of primarily 28 S subunits with an approximate size around 300 kDa (figure 5 and table 2). Another cluster was found that contained five MRP proteins (MRPL10, MRPL12, MRPL45, MRPL53, MRPS21) together with LRPPRC, SLIRP (C14ORF156) and COX7A2 (figure 5 and table 2), with a total approximate molecular mass of about 200 kDa.

**Table 2:** Mitochondrial ribosomal complexes identified by hierarchical clustering of all mitochondrial protein profiles.

*39S mitoribosome*	*28S mitoribosome*	*300 kDa subcomplex*	*200 kDa subcomplex*
MRPL1*	MRPL45*	MRPL18*	MRPS21*
MRPL15*	MRPS10*	MRPS30*	MRPL45*
MRPL17*	MRPS11*	MRPL3*	MRPL53*
MRPL18*	MRPS14*	MRPL2*	MRPL10*
MRPL2*	MRPS16*	MRPS34*	MRPL12*
MRPL21*	MRPS17*	MRPS18B*	*C14ORF156 (SLIRP)*
MRPL22*	MRPS18B*	MRPS22*	*LRPPRC*
MRPL23*	MRPS2*	MRPS27*	*COX7A2*
MRPL24*	MRPS21*	MRPS25*	
MRPL3*	MRPS22*	MRPS17*	
MRPL37*	MRPS23*	**MRPS16**	
MRPL38*	MRPS25*	*SARM1*	
MRPL39*	MRPS27*		
MRPL4*	MRPS28*		
MRPL44*	DAP3 (MRPS29)*		
MRPL49*	MRPS31*		
MRPL9*	MRPS34*		
MRPS30*	MRPS5*		
*AFG3L2*	MRPS7*		
***ICT1***	MRPS9*		
*DBT*	**PTCD3**		
*CAD*	SARM1		
*CLTC*	GPI		
*STOML2*	MIA3		
	*ATAD3A*		
	*DARS*		
	*PHB*		
	*PHB2*		
	FAM82A2		
	EPRS		
	NDUFC2		
	PRDX6		

This table summarizes results from the HCL analysis of all mitochondrial proteins in the dataset. Subunits of the mitoribosome are marked with an asterisk and proven interactors are bold black and underlined. Proteins that were found to co-purify with mitoribosomal proteins in selected affinity purification – mass spectrometry studies are black italic.

### Detailed analysis of mitoribosomal complexes and interactors

Results from the HCL analysis for mitoribosomal proteins prompted us to look at these complexes in more detail with respect to previously reported interacting proteins. All mitochondrial proteins that were previously reported to interact with the mitoribosome based on affinity purification studies by Rorbach [26], Richter [27], and He [24], [25] were exclusively selected for further analysis. Selection of proteins that co-purified with mitoribosomal proteins also provides supportive evidence for putative interactions between co-migrating proteins in our analysis. We focussed on four mitoribosomal complexes of interest: the 28 S cluster, the 39 S cluster, the 300 kDa complex, and the 200 kDa complex. For the refined HCL analysis, we only used data from gel slices that corresponded with the molecular mass region of interest to prevent interference from data of non-relevant gel slices.

**Figure 6:**
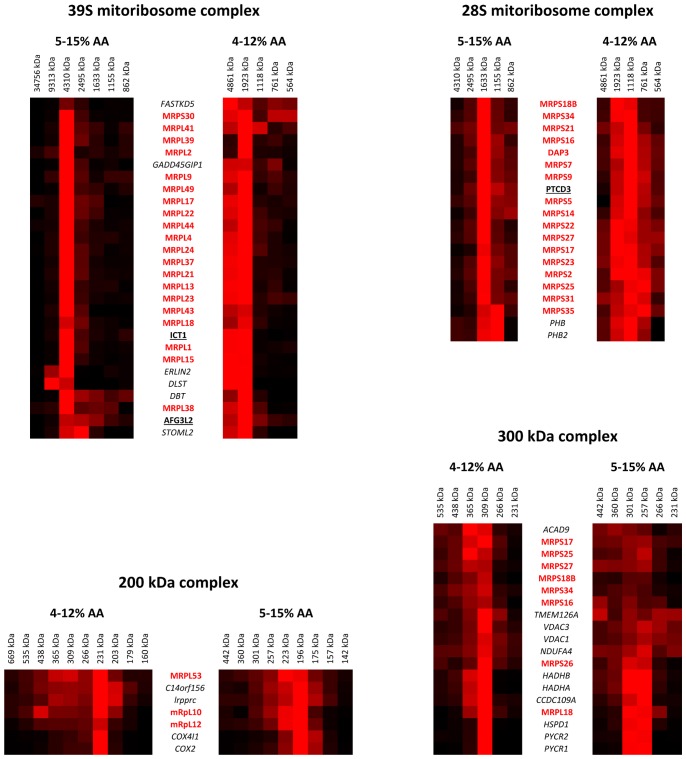
Co-migration of mitoribosomal proteins with previously found interactors in selected affinity purification – mass spectrometry studies. Hierarchical clustering was applied to identify co-migration of previously identified mitoribosome interactors with ribonucleotide complexes. Only gel slices of interest that correspond with the detected mitoribosomal complexes were selected for HCL analysis of each complex to increase sensitivity and specificity of the HCL analysis.

For the analysis of the 28 S and 39 S HCL clusters we selected slices 3–7 and 1–5 from the 4–12% AA and 5–15% AA gels, respectively. The HCL analysis shows the co-migration of eight and ten previously reported interactors with the 28 S and 39 S complexes, respectively. This analysis identified two additional proteins that co-migrate with the 39 S subunits that were not readily identified *via* the HCL analysis of all mitochondrial protein profiles: ERLIN2 and DLST (figure 6 and table 3). Hierarchical clustering analysis for the 300 kDa subcomplex was performed using slices 9–14 and 7–10 of the 4–12% AA and 5–15% AA gels, respectively. The HCL analysis identified eleven additional proteins that co-migrated with seven MRPS and a single MRPL protein (figure 6 and table 3). All of these eleven protein were not picked up in the HCL analysis of all mitochondrial protein profiles. Analysis of the 200 kDa subcomplex by hierarchical clustering was performed using slices 8–18 from the 4–12% AA gel and slices 6–14 from the 5–15% AA gel. Three of the 39 S mitoribosomal subunits apparently co-migrated with C14ORF156 (SLIRP), LRPPRC and two cytochrome *c* oxidase subunits (figure 6 and table 3). This co-migration of cytochrome *c* oxidase subunits with MRP subunits was not observed in the HCL analysis of all mitochondrial protein profiles where the cytochrome c oxidase complex was present in a separate cluster.

**Table 3:** Detected complexes of mitoribosomal proteins together with previously identified interactors by hierarchical clustering.

*39S mitoribosome*	*28S mitoribosome*	*300 kDa subcomplex*	*200 kDa subcomplex*
MRPL1*	MRPL45*	MRPL18*	MRPL10*
MRPL13*	MRPS10*	MRPS16*	MRPL12*
MRPL15*	MRPS11*	MRPS17*	MRPL53*
MRPL17*	MRPS14*	MRPS18B*	*C14ORF156 (SLIRP)*
MRPL18*	MRPS15*	MRPS25*	*LRPPRC*
MRPL2*	MRPS16*	MRPS26*	*COX2*
MRPL21*	MRPS17*	MRPS27*	*COX4L1*
MRPL22*	MRPS18B*	MRPS34*	
MRPL23*	MRPS21*	*ACAD9*	
MRPL24*	MRPS22*	*TMEM126A*	
MRPL37*	MRPS23*	*NDUFA4*	
MRPL38*	MRPS24*	*PYCR1*	
MRPL39*	MRPS25*	*PYCR2*	
MRPL4*	MRPS27*	*VDAC1*	
MRPL41*	DAP3 (MRPS29)*	*VDAC3*	
MRPL43*	MRPS31*	*CCDC109A*	
MRPL44*	MRPS34*	*HADHA*	
MRPL49*	MRPS35*	*HADHB*	
MRPL9*	MRPS5*	*HSPD1*	
MRPS30*	MRPS7*		
*AFG3L2*	MRPS9*		
*DBT*	PHB		
*DLST*	PHB2		
*ERLIN2*	**PTCD3**		
*FASTKD5*			
*GADD45GIP1*			
**ICT1**			
*STOML2*			

Migration profiles from mitochondrial ribosomal proteins and previously identified interactors were subjected to hierarchical clustering analysis to examine possible co-migration. Proteins from the mitoribosome are marked with an asterisk and functionally validated interactors are bold and underlined. Co-purified proteins from selected AP-MS studies are italic.

## Discussion

The complexome profiling dataset provides a valuable resource to identify putative protein-protein interactions or to prioritize proteins of interest on the basis of protein co-migration. Previously, protein co-migration has typically been analyzed by 2D BN SDS-PAGE in combination with western blotting. Technical difficulties associated with immuno detection aside, this approach suffers from low-throughput and high costs when many proteins need to be analyzed. The complexome profiling dataset of this study is essentially the equivalent of 3812 two dimensional BN SDS-PAGE western blot detections (duplicate measurements of 953 proteins from two acryl amide gradient gels) and in addition amenable to computational analysis. This allows simple comparisons of migration profiles between proteins of interest or the use of computer assisted analyses to identify proteins with similar migration profiles. This approach provides independent supportive evidence for protein-protein interactions suggested by other methods (e.g. bioinformatic or co-purification studies) and may thus be used for validation purposes and to prioritize candidate protein-protein interactions for functional validation studies. In addition, complexome profiling data can be used to identify putative protein-protein interactions from an independent and untargeted experiment. However, depending on the research question and setup of the complexome profiling experiment, additional experiments are likely required to prove protein-protein interactions since co-migration of non-interacting proteins may occur.

Here, we used our dataset to analyze co-migration profiles of mitoribosomal proteins and previously described interactors from co-purification studies. By now, several hundred proteins have been suggested to interact with the mitoribosome based on AP-MS studies. These are likely present in multiple complexes which probably include secondary interactions and false positives. As a first step to elucidate these interactions we looked for co-migrating proteins to support alleged interactions. As expected, our analysis showed that the majority of 28 S and 39 S mitoribosomal subunits co-localize in high molecular mass complexes together with previously reported (putative) interactors from AP-MS studies.

For the 28 S and 39 S complexes, eight and three previously reported interactors from AP-MS studies were found to co-migrate, respectively. Of these, two proteins (ICT1, PTCD3) are known *bona fide* interactors or components of the mitoribosome [27], [43], [44]. ICT1 is a functional peptidyl-tRNA hydrolase which has been recruited into the mitochondrial ribosome [27] and PTCD3 has been reported to associate with the small ribosomal subunit to regulate translation [43]. The AFG3L2 protease was found to co-migrate with the 39 S complex in our dataset. This protease is known to regulate assembly of the mitoribosome and biogenesis of its 39 S component [44], which could explain its co-migration with the 39 S complex. The AFG3L2 protein is known to form a heteromeric m-AAA protease complex together with paraplegin [45], which we could not confirm since paraplegin was not available in our dataset. Prohibitin (PHB), prohibitin 2 (PHB2) and STOML2 were found to co-migrate with the 28S and 39 S mitoribosomal complexes. STOML2 is a mitochondrial inner membrane protein of unknown function that is suggested to recruit prohibitins to cardiolipin to form microdomains for optimal assembly of OXPHOS complexes [46], [47]. Interaction of PHB and PHB2 with the mitoribosome was expected as He *et al* reported that both proteins contribute to mitochondrial translation [25]. Interestingly, GADD45GIP1 was recently shown to be essential for synthesis and insertion of mtDNA encoded OXPHOS components [48]. In addition, knockdown of GADD45GIP1 was shown to result in reduced mitochondrial protein synthesis [25]. Collectively, the co-migration and co-purification of these proteins suggests that protein biogenesis and complex assembly are performed in close vicinity. Combined localization of these processes would most likely occur for efficient generation of the inner membrane proteins and complexes. However, further research is required to confirm this hypothesis which is beyond the scope of this paper.

The 300 kDa ribosomal complex is rather difficult to put into context. First of all, several proteins were found to co-migrate which might be part of other complexes of similar size. Both VDAC proteins as well as PYCR1 and PYCR2 are known to form homomeric complexes of about 300 kDa. Similarly, both HADHA and HADHB are components of the hydroxacyl-CoA dehydrogenase complex for which the determined size corresponds with the α2β2 configuration of the enzyme. Interaction of the CCDC109A protein product with the mitoribosome seems unlikely from a biochemical point of view as it encodes the mitochondrial calcium uniporter [49]. ACAD9 and TMEM126B have recently been reported to form a complex I assembly complex together with ECSIT and NDUFAF1 (NDUFAF1 and TMEM126B were not detected in our study) with an approximate size of 300 kDa as determined by complexome profiling [11]. Interestingly, ACAD9 was found here associated with TMEM126A in the 300 kDa complex together with mitoribosomal proteins. Please note that also ECSIT did co-migrate with ACAD9 and TMEM126A in our dataset but was not included in this refined analysis as it was not identified in the affinity purification studies that were used to filter the dataset and is therefore not shown in figure 5. TMEM126A and TMEM126B share an 87% amino acid sequence similarity (32% identity) which, together with its co-migration with ACAD9 and ECSIT, suggests a possible role for TMEM126A in complex I assembly. Detection of NDUFA4 at 300 kDa was rather surprising. This particular protein was recently shown to be a subunit of complex IV rather than complex I [50]. In the same paper the authors show that NDUFA4 contributes to the activity and biogenesis of the holocomplex. In our dataset however, we observed that NDUFA4 was dominantly detected at 300 kDa with only minor abundance at the height of complex I (∼1 MDa) and complex IV (∼200 kDa). This discrepancy might result from the fact that different detergents were used for solubilization between both studies (lauryl maltoside versus digitonin) which requires further research. Interaction of the protein HSPD1 with the 300 kDa ribonucleotide complex might be relevant given its chaperone functionality [51].

Our analyses highlighted a putative 200 kDa ribonucleotide complex that appears to contain three MRPL subunits together with LRPPRC, SLIRP and cytochrome *c* oxidase (COX) subunits. Recent studies demonstrated that LRPPRC and SLIRP interact in a ribonucleoprotein complex involved in posttranscriptional gene expression regulation in mitochondria [52]–[54]. Work of Sasarman *et al.* showed that knockdown of LRPPRC results in an isolated COX deficiency whereas further knockdown of LRPPRC induces a generalized defect of OXPHOS complexes [52]. Co-purification of LRPPRC and SLIRP was confirmed by immunodetection and by 2D BN SDS-PAGE immunoblotting. These analyses showed that LRPPRC and SLIRP form a complex of approximately 250 kDa, which is concordant with our data [52]. The data also showed co-migration of the MRPL proteins with COX subunits (figures 5 and 6) which is presumably caused by overlapping co-localization of both complexes within some of the gel slices. The minimal theoretical mass of a complex that consists of MRPL10, MRPL12, MRPL53, SLIRP and LRPPRC, after removal of mitochondrial transit peptides and assuming 1∶1 stoichiometry for all proteins, is 220 kDa which fits the estimated 200 – 231 kDa mass of the complex in our data. Considering the function of the LRPPRC/SLIRP complex to regulate post transcriptional gene expression one could expect mRNA to be present in this complex. The minimal theoretical mass of the putative MRPL10/MRPL12/MRPL53/SLIRP/LRPPRC complex rules out the possibility of any mitochondrial mRNA to be present in the complex on gel, unless the electrophoretic mobility of the complex is higher than expected. Interestingly, the approximate mass of a complex composed of exclusively LRPPRC (152 kDa), SLIRP (12 kDa) and an average mitochondrial mRNA (300 kDa) is about 460 kDa in theory. Assuming normal electrophoretic mobility of the LRPPRC/SLIRP complex, one would expect additional proteins to be present rather than mRNA with respect to the electrophoretic migration distance of the LRPPRC/SLIRP complex. Nevertheless, further research is required to prove the potential interaction between the MRPL subunits and the previously established LRPPRC/SLIRP complex to rule out the possibility that both complexes co-migrated as non-related, individual complexes.

Our dataset provides the first available analysis of protein complexes in human cells by complexome profiling. The complexome profiling dataset presented in this paper is not exclusively limited to the analysis of mitochondrial protein-protein interactions since a mitochondrial enriched fraction was used. Data for several hundred non-mitochondrial proteins is also available from our dataset that can be used to analyze protein complexes in other cellular compartments. Although this dataset contains a lot of information, future complexome profiling experiments need to be performed in different cell types, tissues, and differential conditions to completely elucidate the composition of mitochondrial protein complexes in humans. Ideally, future complexome profiling datasets should be merged with existing datasets in order to fully understand the complex dynamics of protein-protein interactions. To this end, the fact that protein electrophoretic migration profiles are stored within a data matrix readily facilitates the combination of present and future datasets.

## Conclusions

We have generated a collection of BNE profiles for 953 proteins that can be used to identify novel protein-protein interaction and to underpin or prioritize candidate protein-protein interactions. As an example we have used our dataset to analyze mitoribosomal complexes. Our analysis showed for the first time that mitoribosomal complexes can be analyzed by blue native electrophoresis as demonstrated *via* detection of at least four distinct complexes that are composed of mitoribosomal subunits together with potentially novel and previously reported interactors. In addition, co-migration of multiple proteins associated with the biogenesis of inner membrane complexes together with the 39 S and 28 S mitoribosomal complexes is supportive of co-translational assembly in human cells. Our analysis also exposed a putative 200 kDa ribonucleoprotein complex that potentially contains LRPPRC and SLIRP together with three MRPL subunits. Finally, our data highlights TMEM126A as a putative complex I assembly factor based on co-migration with two known CI assembly chaperones and its sequence similarity with the CI assembly factor TMEM126B which is known to interact with the same two CI assembly factors in other cell types.

## Supporting Information

File S1 **Protein and peptide identification data.**
(XLSX)

File S2 **Distributions of the physicochemical protein properties of the complexome profiling dataset and RefSeq Hs database.**
(PDF)

File S3 **Complexome profiling dataset.**
(XLSX)

File S4 **Overview of complexome profiling data for known complexes in the dataset.**
(PDF)

File S5 **Hierarchical cluster analysis of protein profiles.**
(XLSX)

File S6 **MEV hierarchical cluster analysis file.**
(ZIP)

File S7 **Hierarchical cluster analysis results for the complexome profiling dataset and shuffled matrices.**
(PDF)
