# Supplementary material for: Analysis of 953 Human Proteins from a Mitochondrial HEK293 Fraction by Complexome Profiling
Source: PLoS One. 2013 Jul 23;8(7):e68340. doi: 10.1371/journal.pone.0068340 (PMC3720734; doi:10.1371/journal.pone.0068340)
Supplement: File S2 — Distributions of the physicochemical protein properties of the complexome profiling dataset and RefSeq Hs database. (PDF) [file pone.0068340.s002.pdf]

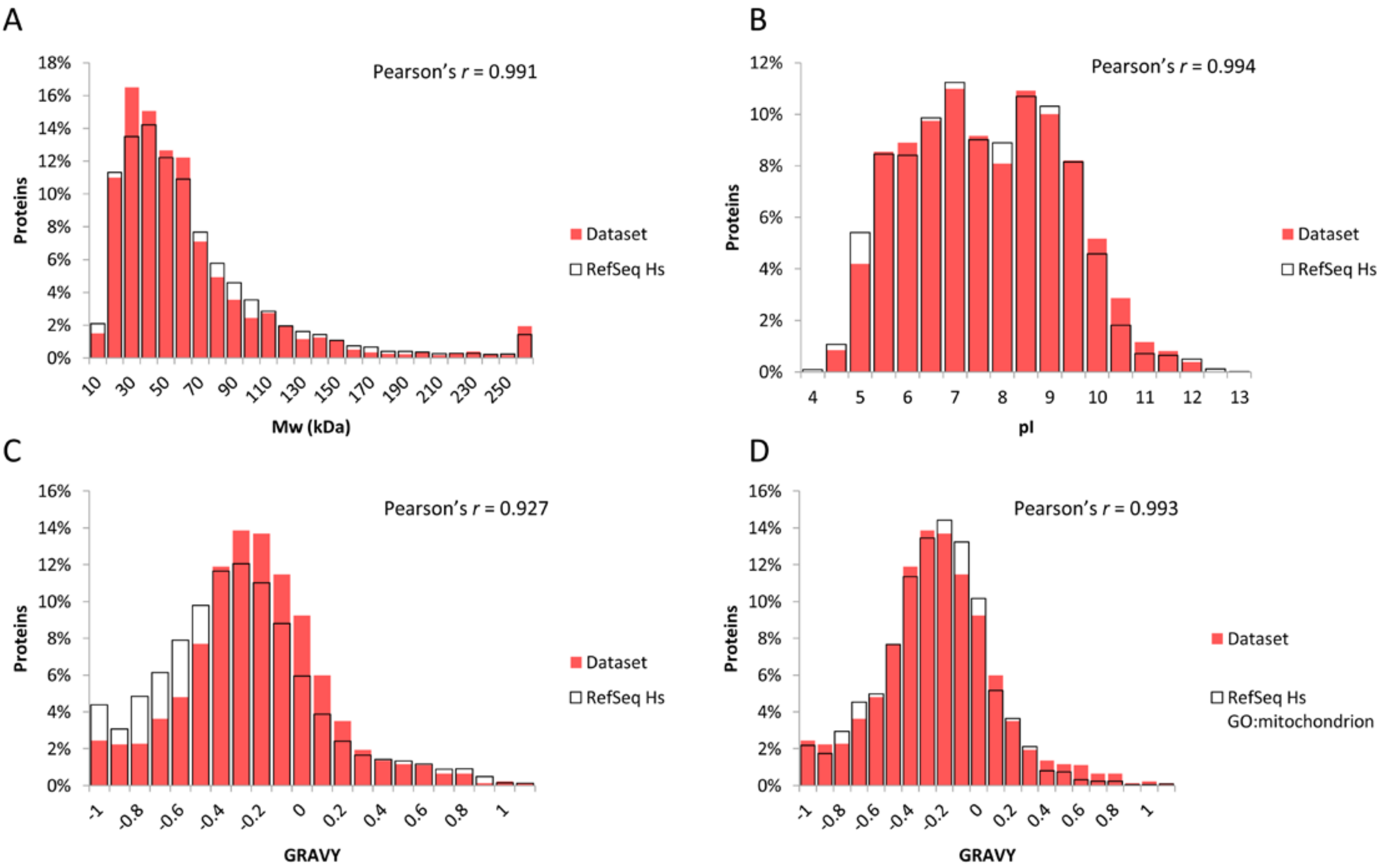

Supplementary figure: Distributions of the physicochemical protein properties of the complexome profiling dataset and RefSeq Hs database. Shown in this figure are the overlaid physicochemical protein distributions from the complexome profiling dataset and the RefSeq Homo sapiens database based on molecular weight (Figure A: Mw), isoelectric point (Figure B: pI) and grand average of hydropathicity (Figure C: GRAVY). Figure D shows the overlaid GRAVY protein distributions of the complexome profiling dataset and all mitochondrial proteins from the RefSeq Hs database (based on gene ontology annotation). Figures A and B show close to identical Mw and pI distributions for both datasets as illustrated by their respective Pearson's correlation coefficients r = 0.991 and r = 0.994. The GRAVY distribution of proteins in the complexome profiling dataset appears to be shifted towards more hydrophobic proteins compared to the distribution of all proteins in the RefSeq Hs database in figure C (Pearson's r = 0.927). To test whether the enriched fraction of mitochondrial proteins could explain this shift we overlaid the GRAVY distribution of our dataset with that of all mitochondrial proteins in the RefSeq Hs database in figure D, which indeed shows a close to identical distribution of protein GRAVY values (Pearson's r = 0.993).
