# Supplementary material for: Analysis of 953 Human Proteins from a Mitochondrial HEK293 Fraction by Complexome Profiling
Source: PLoS One. 2013 Jul 23;8(7):e68340. doi: 10.1371/journal.pone.0068340 (PMC3720734; doi:10.1371/journal.pone.0068340)

Protein	Co-localization 4-12% AA	Co-localization 5-15% AA

Protein migration profiles: 4-12% AA | 5-15% AA

Complex I - NADH dehydrogenase

Protein	4-12% AA	5-15% AA
NDUFA10	+	+
NDUFA11	+	+
NDUFA12	+	+
NDUFA13	+	+
NDUFA2	+	+
NDUFA4	+	+
NDUFA5	+	+
NDUFA7	+	+
NDUFA9	+	+
NDUFB10	+	+
NDUFB4	+	+
NDUFB5	+	+
NDUFB7	+	+
NDUFB9	+	+
NDUFC2	+	+
NDUFV1	+	+
NDUFV2	+	+
NDUFV3	+	+
NDUFS2	+	+
NDUFS3	+	+
NDUFS5	+	+
NDUFS7	+	+
NDUFS8	+	+
NDUFS1	+	+

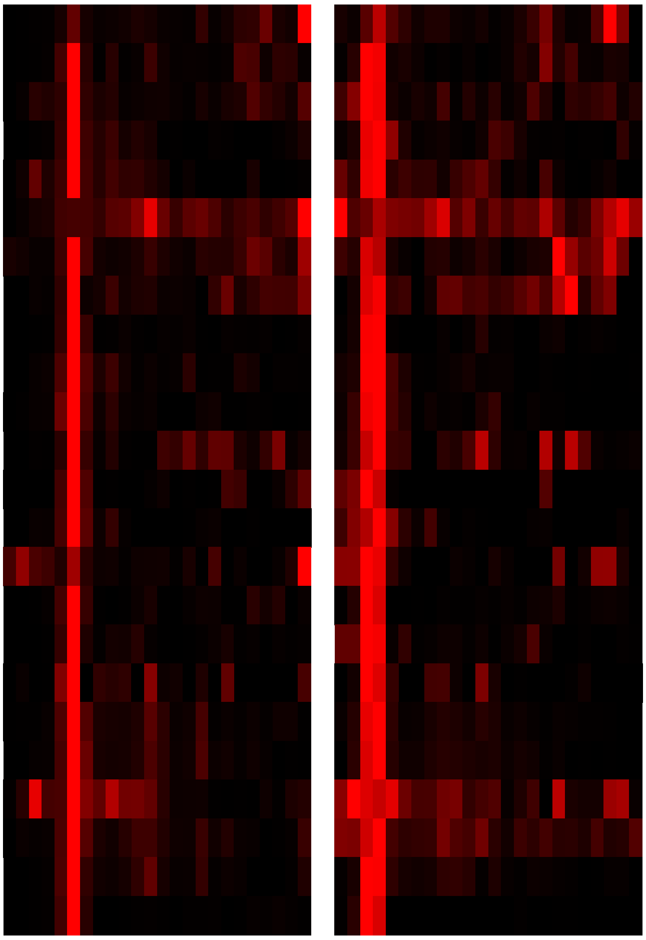

Complex II - Succinate dehydrogenase

Protein	4-12% AA	5-15% AA
SDHA	+	+
SDHB	+	+

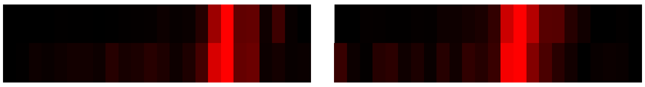

Complex III - Cytochrome bc1 complex

Protein	4-12% AA	5-15% AA
UQCRC1	+	+
UQCRC2	+	+
LOC442454	+	+
UQCRQ	+	+
UQCR10	+	+
UQCRFS1	+	+
CYC1	+	+

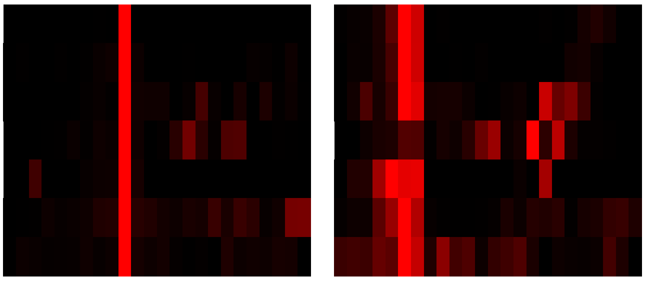

Complex IV - Cytochrome C oxidase

Protein	4-12% AA	5-15% AA
COX4I1	+	+
COX5A	+	+
COX5B	+	+
COX6B1		
COX6C	+	+
COX7A2	+	+
COX2	+	+

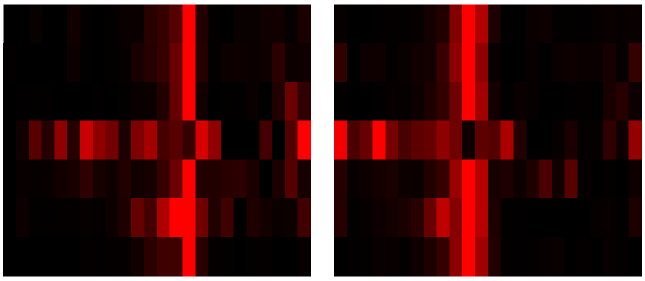

Complex V - ATP synthase

ATP5A1	+	+
ATP5F1	+	+
ATP5B	+	+
ATP5H	+	+
ATP5D	+	+
ATP5I	+	+
ATP5J2	+	+
ATP5L	+	+
ATP5C1	+	+
ATP5O	+	+
ATP5J2-PTCD1	+	+

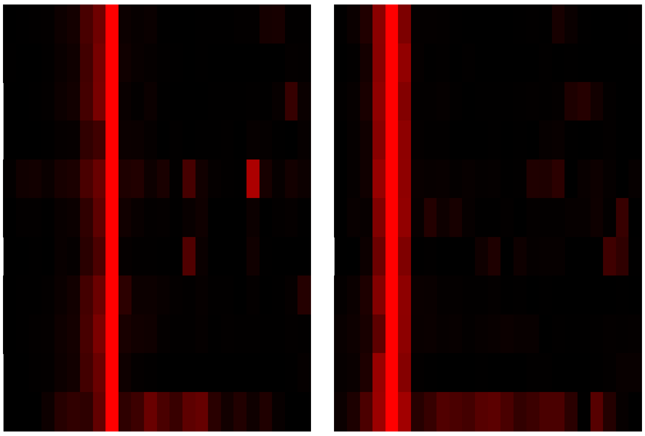

TCP containing chaperone complex

TCP1	+	+
CCT2	+	+
CCT4	+	+
CCT5	+	+
CCT7	+	+
CCT3	+	+
CCT8	+	+
CCT6A	+	+

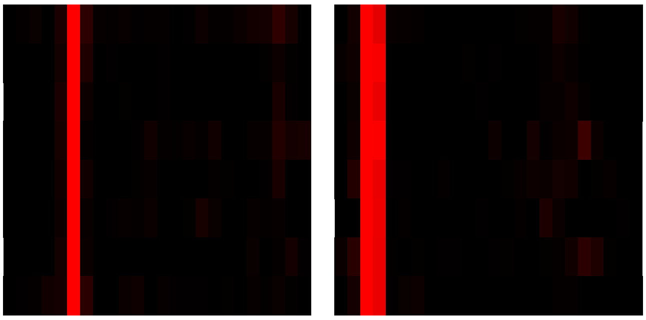

Isocitrate dehydrogenase

IDH3A	+	+
IDH3B	+	+
IDH3B	+	+
IDH3G	+	+

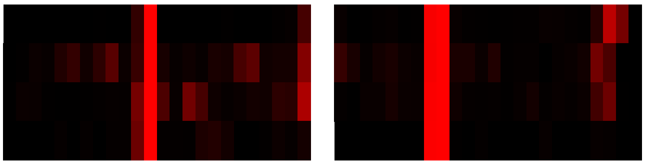

28S mitochondrial ribosome

MRPS10	+	+
MRPS11	+	+
MRPS14	+	+
MRPS15	+	+
MRPS16	+	+
MRPS17	+	+
MRPS18B	+	+
MRPS2	+	+
MRPS21	+	+
MRPS22	+	+
MRPS23	+	+
MRPS24	+	+
MRPS25	+	+
MRPS26	+	+
MRPS27	+	+
MRPS28	+	+
DAP3	+	+
MRPS30		
MRPS31	+	+

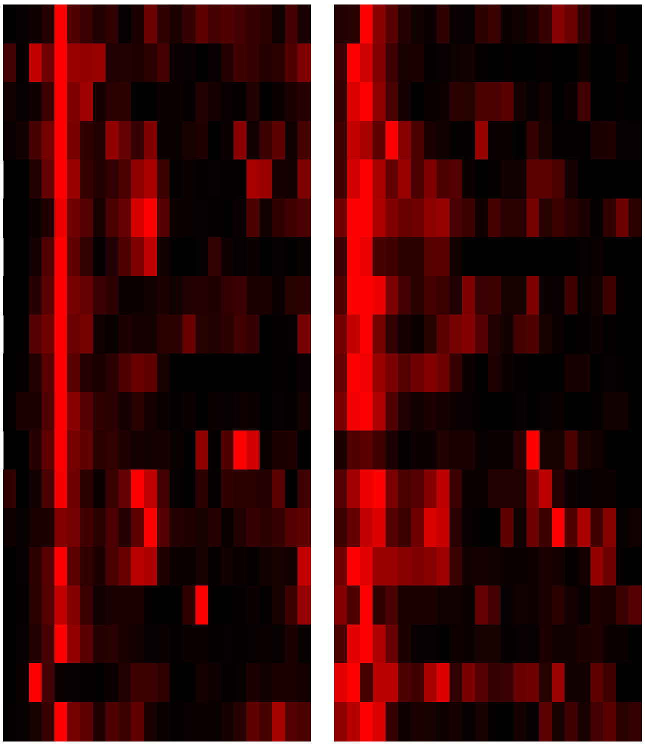

MRPS34	+	+
MRPS35	+	+
MRPS5	+	+
MRPS7	+	+
MRPS9	+	+

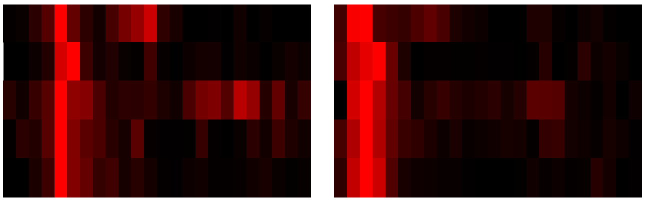

39S mitochondrial ribosome

MRPL1	+	+
MRPL10		
MRPL11		
MRPL12		
MRPL13	+	+
MRPL14		
MRPL15	+	+
MRPL17	+	+
MRPL18	+	+
MRPL19	+	+
MRPL2	+	+
MRPL21	+	+
MRPL22	+	+
MRPL23	+	+
MRPL24	+	+
MRPL3	+	+
MRPL37	+	+
MRPL38	+	+
MRPL39	+	+
MRPL4	+	+
MRPL41	+	+
MRPL43	+	+
MRPL44	+	+
MRPL45	+	+
MRPL46		
MRPL48		
MRPL49	+	+
MRPL53		
MRPL9	+	+

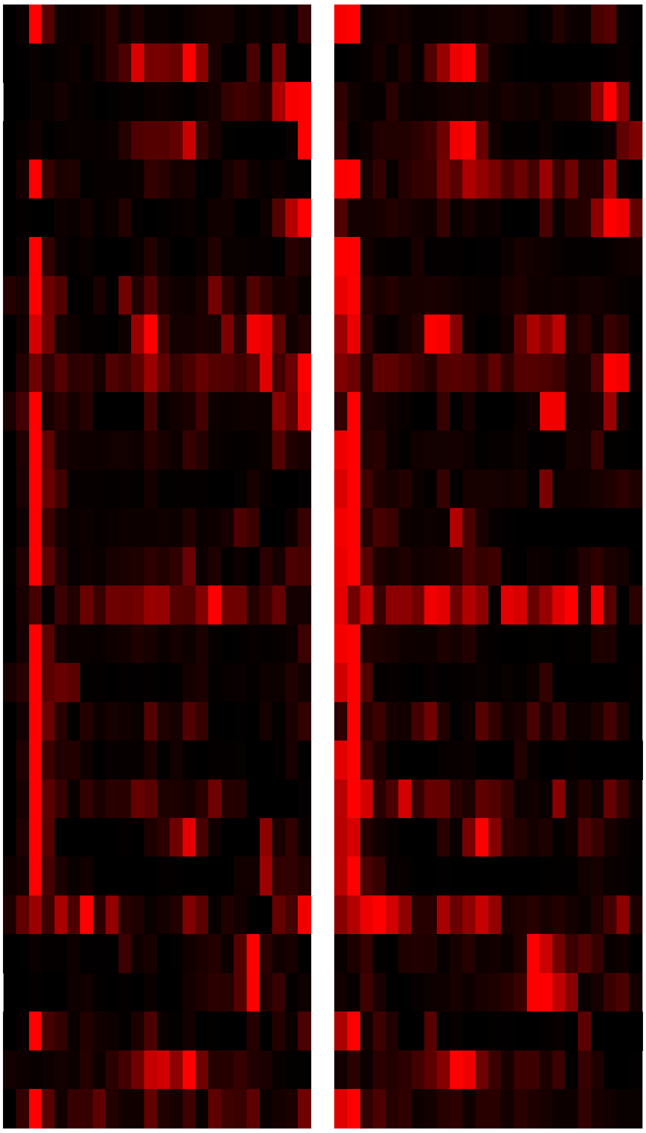

Pyruvate dehydrogenase

PDHA1		
PDHB		
PDPR		

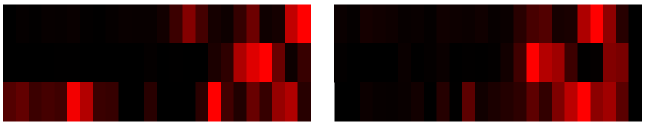

propionyl-CoA carboxylase

PCCA	+	+
PCCB	+	+

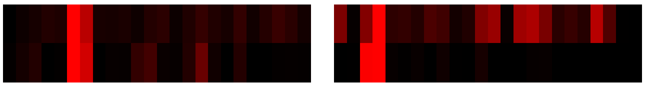

Prohibitin complex

PHB	+	+
PHB2	+	+

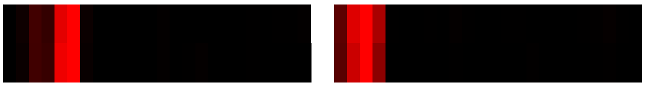

2-oxoisovalerate dehydrogenase

BCKDHA	+	+
BCKDHB	+	+

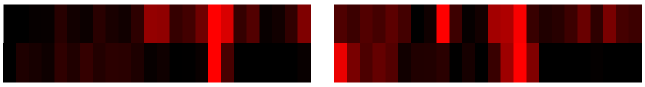

electron transfer flavoprotein

ETFA	+	+
ETFB	+	+

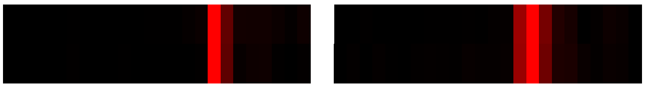

Trifunctional enzyme

HADHA	+	+
HADHB	+	+

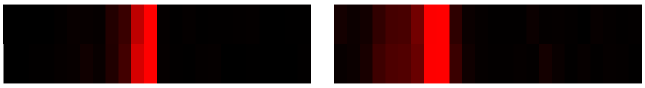

Non-mitochondrial complexes

Proteasome

PSMA3	+	+
PSMA6	+	+
PSMA7	+	+
PSMB1	+	+
PSMB3	+	+
PSMB4	+	+
PSMB5	+	+

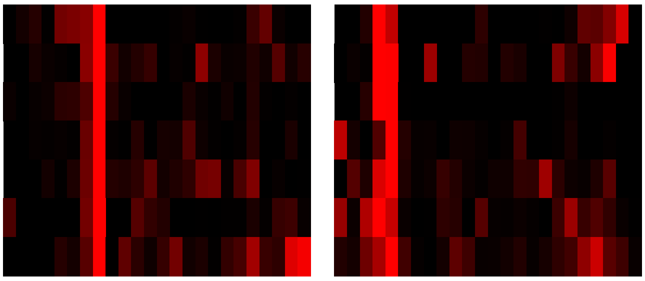

Integrin complex

ITGA1	+	+
ITGA2	+	+
ITGA5	+	+
ITGA6	+	+
ITGAV	+	+
ITGB1	+	+

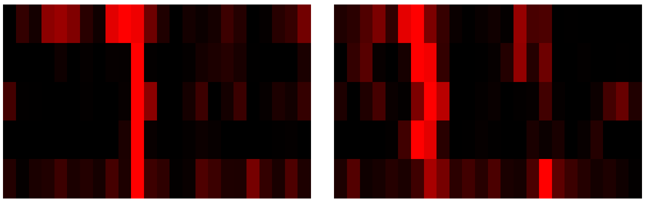

V-type proton ATPase: V1 part

ATP6V1A	+	+
ATP6V1B2	+	+
ATP6V1E1	+	+

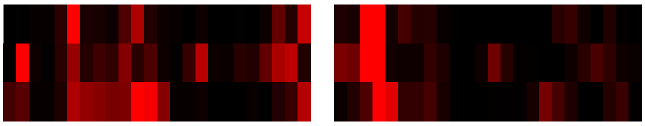

V-type proton ATPase: V0 part

ATP6AP1	+	+
ATP6V0A2	+	+
ATP6V0D1	+	+

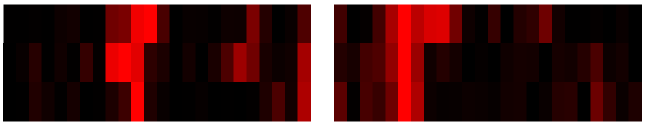

dolichyl-diphosphooligosaccharide--protein glycosyltransferase

DDOST	+	+
RPN1	+	+
RPN2	+	+
DAD1	+	+
STT3A	+	+
STT3B		

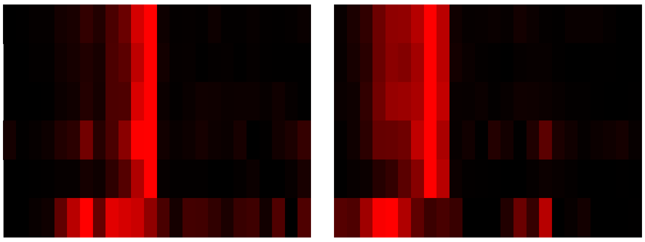

beta-hexosaminidase

HEXA	+	+
HEXB	+	+

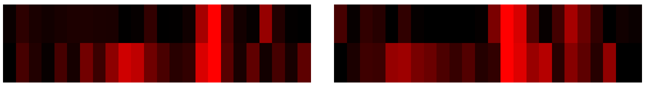

40S Ribosome

RPS13	+	+
RPS15A		+
RPS16	+	+
RPS18	+	
RPS19	+	
RPS2	+	+
RPS24	+	+
RPS3	+	
LOC100130107		
RPS4X	+	+
RPS5	+	+
RPS6	+	+
RPS7	+	
RPS8	+	+
RPS9	+	+
RPSA		

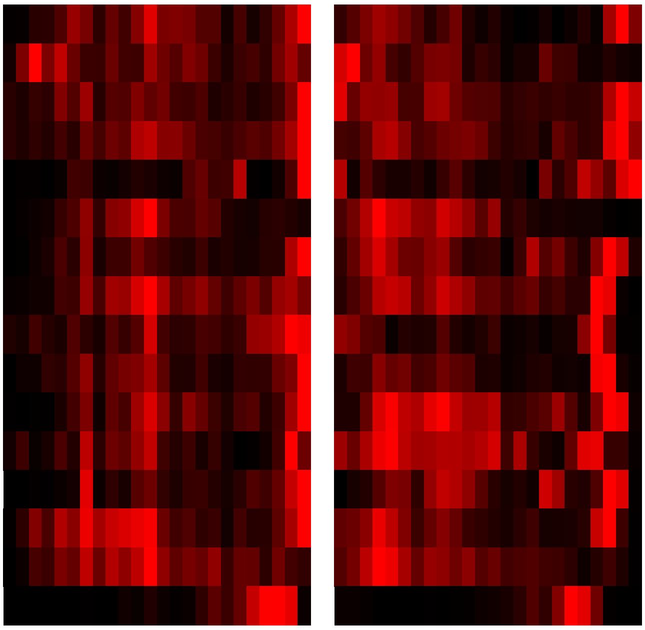

60S Ribosome

RPLP0	+	+
RPLP2		+
RPL10	+	+
RPL10A		
RPL11		+
RPL12		+
RPL13	+	+
RPL13A	+	+
RPL14	+	+
RPL15	+	+
LOC729046	+	+
RPL18	+	+
RPL18A	+	+
RPL19	+	+
RPL21	+	+
RPL22		
RPL23		
RPL24	+	+
RPL26		+
RPL27A	+	+
LOC653881	+	+
RPL30		+
RPL31		
RPL32		+
RPL35A	+	+
RPL4	+	+
RPL5		

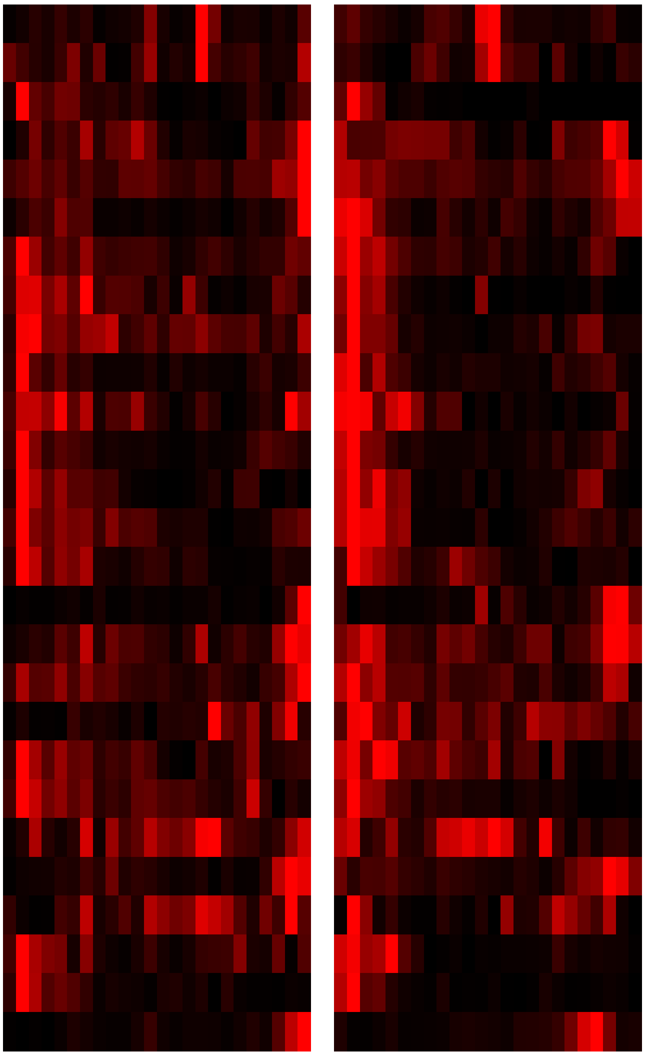

RPL6	+	+
RPL7	+	+
RPL7A	+	+
RPL8		+
RPL9		

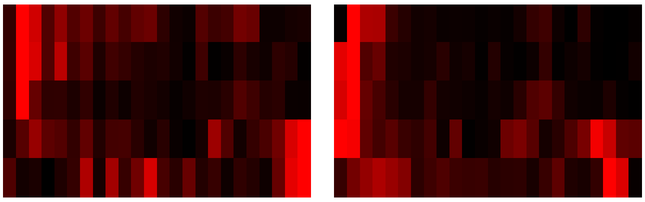

Supplement: File S4 — Overview of complexome profiling data for known complexes in the dataset. (PDF) [file pone.0068340.s004.pdf]
