# Supplementary material for: Analysis of 953 Human Proteins from a Mitochondrial HEK293 Fraction by Complexome Profiling
Source: PLoS One. 2013 Jul 23;8(7):e68340. doi: 10.1371/journal.pone.0068340 (PMC3720734; doi:10.1371/journal.pone.0068340)
Supplement: File S7 — Hierarchical cluster analysis results for the complexome profiling dataset and shuffled matrices. (PDF) [file pone.0068340.s007.pdf]

Supplementary table: Clustering results for the complexome dataset and shuffled matrices

		Complexome dataset		Shuffled Dataset (n=10)	
**Protein complex**	**Detected subunits**	**Clusters**	**Co-clustered subunits**	**Clusters**	**Co-clustered subunits**
Complex I - NADH dehydrogenase	28	1	22	1	2
Complex II - Succinate dehydrogenase	2	1	2	0	0
Complex III - Cytochrome bc1 complex	7	1	6	0	0
Complex IV - Cytochrome C oxidase	7	1	5	0	0
Complex V - ATP synthase	11	1	10	0	0
28S mitochondrial ribosome	24	3	22	2.5	5.5
2-oxoglutarate dehydrogenase	3	0	0	0	0
2-oxoisovalerate dehydrogenase	2	1	2	0	0
39S mitochondrial ribosome	29	2	20	2	4
electron transfer flavoprotein	2	1	2	0	0
Isocitrate dehydrogenase	4	1	4	0	0
Prohibitin complex	2	1	2	0	0
propionyl-CoA carboxylase	2	1	2	0	0
Pyruvate dehydrogenase	3	0	0	0	0
TCP containing chaperone complex	8	1	7	0	0
Trifunctional enzyme	2	1	2	0	0

Protein complex: mitochondrial protein complex description, **detected subunits:** number of subunits for which profiles could be constructed in both gradients, **clusters:** number of clusters in the hierarchical cluster analysis that contains two or more subunits of a respective complex**, co-clustered subunits:** total number of subunits that group together in one or more clusters.
